# Dr. W. W. H. Thackston

**Published:** 1870-09

**Authors:** 


*Dr, W. W. H. Thackston.*—We are pleased to learn that this gentleman, one of the most eminent practitioners of dentistry in the South, has been elected Mayor of Farmville, Virginia, where he has long resided. Dr. Thackston is one of the early graduates of the Baltimore College of Dental Surgery, of the class of 1842.

